# Clinical-epidemiological profile of infantile-juvenile head and neck cancer: a descriptive hospital-based study

**DOI:** 10.1590/1984-0462/2025/43/2023205

**Published:** 2025-10-27

**Authors:** Alessandra Laís Pinho Valente Pires, Lísia Daltro Borges Alves, Dawid Vinicius Rios do Ouro, Samille Marques Fontoura de Oliveira, Cristiane Brandão Santos Almeida, Adriana Mendonça da Silva, Valéria Souza Freitas

**Affiliations:** a Universidade Estadual de Feira de Santana – Feira de Santana (BA), Brazil.; b Instituto Nacional de Câncer, Divisão de Pesquisa Clínica – Rio de Janeiro (RJ), Brazil.; c Unidade de Ensino Superior de Feira de Santana – Feira de Santana (BA), Brazil.

**Keywords:** Squamous cell carcinoma, Risk factors, Kids, Teenagers, Carcinoma de células escamosas, Fatores de risco, Crianças, Adolescentes

## Abstract

**Objective:**

To describe the clinical-epidemiological profile of infantile-juvenile individuals diagnosed with head and neck cancer in Northeast Brazil from 1985 to 2017.

**Methods:**

Descriptive study based on data collected from the Brazilian Hospital Cancer Registry System. This study included all individuals diagnosed with head and neck cancer, aged between zero and 19 years, with records in the Federative Units of Northeast Brazil, from 1985 to 2017. The collected variables included sociodemographic characteristics, risk factors, and tumor’s clinical characteristics. Frequency tables with respective percentages were used for qualitative variables while descriptive measures such as mean and standard deviation were adopted for quantitative variables. Data were descriptively analyzed using the software Statistical Package for the Social Sciences (SPSS).

**Results:**

There were 500 cases of pediatric head and neck cancer, with more cases registered in Bahia (31.8%). The mean age of the individuals was 13.7±4.3 years. The majority were male (58.0%), non-white (80.4%), with incomplete elementary education (57.8%), and without a partner (94.2%). Most of them were non-smokers (70.3%), non-alcohol consumers (68.6%), and did not have a family history of cancer (66.8%). The most frequent histological type was squamous cell carcinoma (32.1%). Tumors were predominantly located in the nasopharynx (52.4%), staged as T4 (36.3%), N0 (40.7%), and M0 (90.9%).

**Conclusions:**

Cases of head and neck cancer in the infantile-juvenile population were predominantly found in individuals from Bahia, male, aged between 12 and 16 years, of non-white ethnicity, incomplete elementary education degree, without a partner, non-smokers, non-drinkers, and without a family history of cancer. The most affected anatomical region was the nasopharynx, with the most frequent histological type being squamous cell carcinoma.

## RESUMO

**Objetivo:**

Descrever o perfil clínico-epidemiológico de indivíduos infantojuvenis diagnosticados com câncer de cabeça e pescoço no nordeste do Brasil de 1985 a 2017.

**Métodos:**

Estudo descritivo baseado em dados coletados do Sistema de Registro Hospitalar de Câncer do Brasil. Este estudo incluiu todos os indivíduos diagnosticados com câncer de cabeça e pescoço, com idades entre zero e 19 anos, com registros nas Unidades Federativas do nordeste do Brasil, de 1985 a 2017. As variáveis coletadas incluíram características sociodemográficas, fatores de risco e características clínicas do tumor. Foram utilizadas tabelas de frequência com respectivos percentuais para variáveis qualitativas e medidas descritivas como média e desvio padrão para variáveis quantitativas. Os dados foram analisados descritivamente por meio do *software* Statistical Package for the Social Sciences (SPSS).

**Resultados:**

Foram identificados 500 casos de câncer de cabeça e pescoço pediátrico, com mais casos registrados na Bahia (31,8%). A idade média dos indivíduos foi de 13,7 (±4,3) anos. A maioria era do sexo masculino (58,0%), não branca (80,4%), com nível fundamental incompleto (57,8%) e sem companheiro(a) (94,2%). A maioria deles não fumava (70,3%), não consumia álcool (68,6%) e não tinha histórico familiar de câncer (66,8%). O tipo histológico mais frequente foi o carcinoma de células escamosas (32,1%). Os tumores foram predominantemente localizados em nasofaringe (52,4%), classificados como T4 (36,3%), N0 (40,7%) e M0 (90,9%).

**Conclusões:**

Os casos de câncer de cabeça e pescoço na população infantojuvenil foram encontrados predominantemente em indivíduos baianos, do sexo masculino, com idade entre 12 e 16 anos, de etnia não branca, ensino fundamental incompleto, sem companheiro, não fumantes, não etilistas e sem histórico familiar de câncer. A região anatômica mais acometida foi a nasofaringe, sendo o tipo histológico mais frequente o carcinoma espinocelular.

## INTRODUCTION

 Despite leukemia, central nervous system tumors, and lymphomas being the most frequent neoplasms in the infantile-juvenile population, studies have indicated an increased incidence of head and neck tumors in pediatric patients, including squamous cell carcinoma (SCC), adenocarcinoma, lymphoma, rhabdomyosarcoma (RMS), neuroblastoma, among others.^1-3^ Currently, head and neck cancer is the sixth most common cancer worldwide, accounting for approximately 2 to 15% of all cancer cases affecting the infantile-juvenile population.^4,5^

 Head and neck cancer is considered a public health problem due to its severity, mortality rate, and high financial cost involved from diagnosis to treatment.^6^ Infantile-juvenile head and neck cancer can exhibit faster growth and greater invasion capacity than that affecting adults, representing a significant cause of infantile-juvenile morbidity and mortality.^7^ In developed countries, it is the second leading cause of death by disease at five to nine years.^8^ Despite that, pediatric individuals tend to respond better to treatment, thus presenting a better prognosis, with approximately 80% cure rate when diagnosed early and treated at specialized centers.^9^

 Epidemiological studies on the clinical-pathological profile of children and adolescents affected by head and neck cancer are scarce in the Brazilian population, and there are no published data on this issue regarding the Northeast region.^10^ Therefore, the objective of this study is to describe the clinical-epidemiological profile of infantile-juvenile individuals diagnosed with head and neck cancer in Northeast Brazil from 1985 to 2017, based on data from the Brazilian Hospital Cancer Registry System. 

## METHOD

 This is a descriptive study based on secondary data collected from the Brazilian Hospital Cancer Registry System (SisRHC). This is a national, open-access health information system consolidated by the Integrated Module of Hospital Cancer Records ( https://irhc.inca.gov.br), that gathers information from all Brazilian Hospital Cancer Registries (HCR). Access to SisRHC information can be obtained through TabNet, a technology developed by the Department of Informatics of the Unified Health System — DATASUS ( https://datasus.saude.gov.br/informacoes-de-saude-tabnet/). 

 This study included cases of all individuals diagnosed with head and neck cancer (ICD C00-14), aged between zero and 19 years, with records in the Federative Units of the Northeast region of Brazil, from 1985 to 2017. Cases with other topographic locations, those lacking histopathological confirmation, or those outside the determined period and age range were not included in this study. 

 The collected variables included sociodemographic characteristics (age, sex, race/skin color, education, marital status, and region of residence), risk factors (smoking, alcohol consumption, and family history), and tumor’s clinical characteristics (location, histological type, initial staging, treatment, and survival status). The race/skin color variable was dichotomized into white and non-white (yellow, brown, black, and indigenous). The initial staging was evaluated using the TNM classification, where ’T’ represents the size of the primary tumor, ’N’ represents lymph node metastases, and ’M’ represents distant metastases. 

 The obtained data were descriptively analyzed using the program Statistical Package for the Social Sciences (SPSS) version 17.0 (SPSS Inc., Chicago, IL, USA). Frequency tables with respective percentages were used for qualitative variables while descriptive measures such as mean and standard deviation were adopted for quantitative variables. 

 This study waived the need for consideration by the Ethics Committee for Research on Human Beings (CEP), considering that the data used are from an open access Brazilian information system, available online, in which the data are presented without identifying the subjects. 

## RESULTS

**Figure 1:**
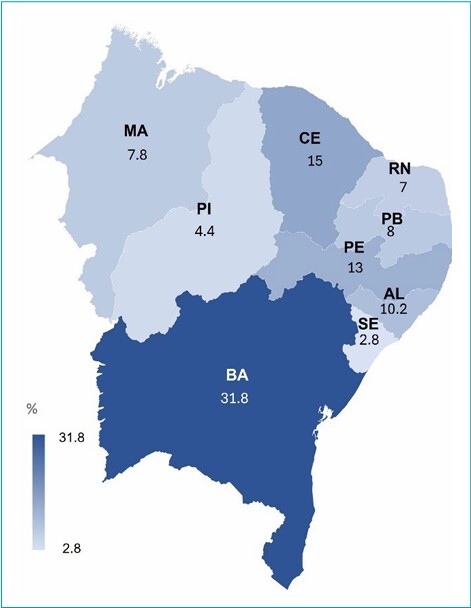
Frequency of infantile-juvenile head and neck cancer at Brazilian Northeast Federative Units, from 1985 to 2017, according to the Brazilian Hospital Cancer Registry System.

 In the Northeast region of Brazil, there were 500 cases of head and neck cancer identified in pediatric patients registered in the SisRHC between 1985 and 2017. The Federal Unit with the highest number of cases was Bahia (31.8%), followed by Ceará (15.0%) (Figure 1). 

 The mean age of the individuals was 13.7±4.3 years, with the highest number of cases in the age group of 12–16 years (39.8%). The majority of the individuals were male (58.0%), nonwhite (80.4%), with incomplete elementary education (57.8%), and without a partner (94.2%). Moreover, most of them were non-smokers (70.3%), non-alcohol consumers (68.6%), and did not have a family history of cancer (66.8%) (Table 1). 

**Table 1:** Sociodemographic characteristics and risk factors of infantile-juvenile individuals diagnosed with head and neck cancer in Northeast Brazil, from 1985 to 2017, according to the Brazilian Hospital Cancer Registry System.

Variable		n	%
Age (years) (n=500)			
	0≥5	32	6.4
	6≥11	101	20.2
	12≥16	199	39.8
	17≥19	168	33.6
Sex (n=500)			
	Male	290	58.0
	Female	210	42.0
Race/Ethnicity (n=428)^*^			
	Non-white	344	80.4
Educational level (n=344)^†^			
	Illiterate	21	6.1
	Elementary education (incomplete)	199	57.8
	Elementary education (complete)	71	20.6
	High school (complete)	50	14.5
	Undergraduate degree (incomplete)	2	0.6
	Undergraduate degree (complete)	1	0.3
Civil status (n=450)^‡^			
	Without partner	424	94.2
Smoking (n=316)^§^			
	Smoker	19	6.0
	Non-smoker	222	70.3
	Not applicable	75	23.7
Alcohol consumption(n=318)^//^			
	Yes	24	7.5
	No	218	68.6
	Not applicable	76	23.9
Family history of cancer (n=208)^¶^			
	No	139	66.8

* 72 missing data

† 156 missing data

‡ 50 missing data

§ 184 missing data

// 182 missing data

¶ 292 missing data.

 The most frequent histological type was SCC (32.1%), followed by undifferentiated carcinoma (15.7%) (Table 2). Tumors were predominantly diagnosed in the nasopharynx (52.4%), staged as T4 (36.3%), N0 (40.7%), and M0 (90.9%) (Table 3). The most employed treatment was radiotherapy combined with chemotherapy (21.7%), followed by isolated chemotherapy (17.8%). Regarding the survival status, 82.2% of the individuals were alive, while 1.60% had deceased (Table 4). 

**Table 2:** Histological type of infantile-juvenile individuals diagnosed with head and neck cancer in Northeast Brazil, from 1985 to 2017, according to the Brazilian Hospital Cancer Registry System.

Histologic type (n=496)	n	%
Adenocarcinoma	15	3.0
Acinic cell carcinoma	13	2.6
Squamous cell carcinoma	159	32.1
Undifferentiated carcinoma	78	15.7
Lymphoepithelial carcinoma	35	7.1
Mucoepidermoid carcinoma	43	8.7
Carcinoma not specified	22	4.4
Burkitt lymphoma	16	3.2
Mature B-cell lymphoma	9	1.8
Hodgkin lymphoma	6	1.2
Non-Hodgkin lymphoma not specified	13	2.6
Histiocytosis neoplasia	5	1.0
Others^*^	13	2.6
Rhabdomyosarcoma	35	7.1
Fusocellular sarcoma	4	0.8
Undefined tumor	2	0.4
Unspecified malignant tumor	25	5.0
Malignant mixed tumor, salivary gland type	3	0.6

* 1 case each of: Fibrosarcoma, Gliomas, Malignant Hemangiopericytoma, Malignant Hemangiopericytoma, Marginal Zone B-Cell Lymphoma, Lymphoblastic Lymphoma, Malignant Lymphoblastic Lymphoma, Malignant Melanoma, Malignant Myoepithelioma, Sarcoma, Soft Tissue Sarcoma, Giant Cell Tumor, Yolk Sac Tumor, and Malignant Fusiform Cell Tumor.

**Table 3:** Tumor characteristics of infantile-juvenile individuals diagnosed with head and neck cancer in Northeast Brazil, from 1985 to 2017, according to the Brazilian Hospital Cancer Registry System.

Variable		n	%
Primary tumor location (n=500)			
	C00 Lip	7	1.4
	C02 Tongue	4	0.8
	C03 Gum	5	1.0
	C04 Floor of the mouth	6	1.2
	C05 Palate	32	6.4
	C06 Other and unspecified parts of mouth	22	4.4
	C07 Parotid gland	83	16.6
	C08 Other major salivary glands	20	4.0
	C09 Tonsil	15	3.0
	C10 Oropharynx	17	3.4
	C11 Nasopharynx	262	52.4
	C12 Pyriform sinus	1	0.2
	C13 Hypopharynx	2	0.4
	C14 Other and ill-defined sites in the lip, oral cavity, and pharynx	12	2.4
T Stage (n=171)^*^			
	T1	26	15.2
	T2	43	25.1
	T3	40	23.4
	T4	62	36.3
N Stage (n=177)^†^			
	N0	72	40.7
	N1	19	10.7
	N2	43	24.3
	N3	43	24.3
M Stage (n=165)^‡^			
	M0	150	90.9
	M1	15	9.1

* 329 missing data

† 323 missing data

‡ 335 missing data

**Table 4:** Treatment and survival status of infantilejuvenile individuals diagnosed with head and neck cancer in Northeast Brazil from 1985 to 2017, according to the Brazilian Hospital Cancer Registry System.

Variable		n	%
Treatment type (n=494)^*^			
	Surgery	76	15.4
	Surgery and chemotherapy	36	7. 3
	Surgery and radiation therapy	29	5.9
	Surgery, radiation therapy, and chemotherapy	38	7.7
	None	40	8.1
	Other	7	1.4
	Chemotherapy	88	17.8
	Radiotherapy	72	14.6
	Radiation therapy and chemotherapy	107	21.7
	Radiation therapy, chemotherapy, and immunotherapy	1	0.2
Survival status (n=500)			
	Death	8	1.6
	Refusal of treatment	1	0.2
	Treatment performed outside	11	2.2
	Advanced disease, lack of clinical conditions, or other associated diseases	2	0.4
	Abandonment of treatment	2	0.4
	Other reasons	35	7.0
	Alive	411	82.2
	No information	30	6.0

* 6 missing data.

## DISCUSSION

 The present study identified 500 cases of head and neck cancer in the Northeast in individuals aged zero to 19 years, between 1985 and 2017. Bahia represented the Federative Unit with the highest number of cases, which may be related to its higher populational coefficient^11,12^ and/or a greater number of specialized centers for pediatric oncological treatment compared to the others in this region. Currently, Bahia has 15 High Complexity Oncology Units (UNACON) and one High Complexity Oncology Center (CACON) distributed among eight cities.^11^

 The mean age of pediatric patients with head and neck cancer found was 13 years, with the age group of 12–16 years being the most predominant. Global evidence indicates that pediatric patients between 10–19 years are more susceptible to developing this type of cancer, with an age variation being possible as some types of tumors are more incident in certain age groups.^13^

 Infantile-juvenile male individuals were more affected by head and neck cancer, a finding also reported by previous studies.^2 ,5,14-17^ A systematic review found that the male gender was more prevalent in approximately 60% of head and neck cancer cases in pediatric patients worldwide.^13^ However, a higher frequency of females was observed in a study conducted in Brazil.^18^

 Regarding ethnicity, a higher number of non-white individuals was observed. While some studies conducted in Brazil corroborate this finding,^18,19^ another one found a higher frequency among white individuals.^14^ Variations in the ethnic profile of pediatric patients with head and neck cancer in this country can be attributed to the population’s miscegenation, involving European, African, and Amerindian descendants, which influences genetic susceptibility and diseases.^20^

 Pediatric patients with head and neck cancer did not have a partner and had incomplete elementary education, which is plausible since these are young patients in school age. The fact that these patients were non-smokers or alcohol consumers and did not have a family history of cancer may indicate a greater genetic involvement in the development of malignant neoplasms in this population, even though only 10% of these tumors are related to a genetic predisposition.^13,21^

 The anatomical head and neck region most affected by cancer in this study was the nasopharynx, corroborating Arboleda et al.^22^ Although the frequency varies depending on each geographic region, lymphomas, carcinomas, and sarcomas are the most common histological origin affecting the head and neck region in children and adolescents.^22^ A recent systematic review showed that male patients older than ten years of age were most affected by lymphomas.^13^ However, in this study, the most frequent histological type was SCC. Findings from other studies also identified carcinoma as one of the most frequent histopathological subtypes of head and neck cancer in young individuals in Brazil.^14^

 The results presented here regarding the initial staging showed a majority of locally advanced tumors (T4), without lymph node (N0) or distant metastases (M0), treated with a combination of radiotherapy and chemotherapy, and they were alive after treatment, similar to other findings.^19 ,22^

 Among the limitations of the study, we can highlight the use of secondary data, which are subject of underreporting, as well as possible filling or structural problems in maintaining the RHC. The need to sensitize healthcare professionals for the proper filling of medical records should be emphasized for a better understanding of individuals’ medical history. Furthermore, as a limitation of the study design,^23^ the results presented here do not allow associations of causal inferences between the analyzed variables and the etiology of infantile-juvenile head and neck cancer. 

 This study provides crucial data in characterizing the profile of this population that can contribute to the development of public policies for the diagnosis and treatment of this specific population.^10^ Additionally, it is important for further studies to be conducted to better investigate the factors associated with the development of head and neck cancer in young individuals, contributing primarily to its prevention. 

 In conclusion, cases of head and neck cancer in infantile-juvenile individuals diagnosed in the Northeast between 1985 and 2017 were predominantly found in individuals from Bahia, male, aged between 12 and 16 years, of non-white ethnicity, with incomplete elementary education degree, without a partner, non-smokers, non-drinkers, and without a family history of cancer. The most affected anatomical region was the nasopharynx, with the most frequent histological type being SCC. The initial staging showed a majority of locally advanced tumors (T4), without lymph node (N0) or distant metastases (M0), treated with a combination of radiotherapy and chemotherapy, with patients alive after treatment. The findings presented made it possible to know the clinical-epidemiological profile of infantile-juvenile individuals diagnosed with head and neck in Northeast Brazil, helping to direct some prevention and care actions and point out the need for analytical studies to assess a possible association between risk factors and pediatric head and neck cancer. 

## Data Availability

The database that originated the article is available with the corresponding author.
